# Three-dimensional secondary reconstruction of mistreated zygomatic fractures using patient specific surgical guides and implants

**DOI:** 10.3389/froh.2024.1464012

**Published:** 2024-09-19

**Authors:** Andrei Krasovsky, Ahmad Hija, Nidal Zeineh, Amir Bilder, Omri Emodi, Adi Rachmiel, Dekel Shilo

**Affiliations:** ^1^ Department of Oral and Maxillofacial Surgery, Rambam Health Care Campus, Haifa, Israel; ^2^ Ruth & Bruce Rappaport Faculty of Medicine at the Technion-Israel Institute of Technology, Haifa, Israel

**Keywords:** zygoma, malar, refracture, 3D, patient specific implant, surgical guide, 3D printing, 3D planning

## Abstract

**Introduction:**

The zygomatic bone has a great impact on the anterior and lateral projection of the midface as well as the proper position of the globe. Primary alignment of zygomatic fractures is very important as secondary reconstruction is far more challenging. Treatment of misaligned zygoma requires refracturing of the bone to allow for repositioning. Due to the great impact of the zygoma on the projection of the midface, a precise 3D realignment is of great importance. Technology nowadays develops rapidly and allows for superior results in many surgical fields. The use of patient specific surgical guides and fixation plates is becoming more abundant.

**Methods:**

Using 3D segmentation and design software, we developed a sequence for using 3D planning and printing both for the refracturing stage, avoiding a coronal approach, and for precise repositioning and fixation of the zygoma in the new position.

**Results:**

The method is described as well as a unique advanced 3D analysis, allowing for objectively assessing the results. Two cases are presented, including the design and post operative changes.

**Discussion:**

Pre-op, planned and final positions were compared and showed exceptional accuracy allowing for the elimination of human errors which are common in a 3D sensitive procedure such as refracturing of the zygoma. This method can easily be applied to other secondary reconstruction procedures requiring realignment.

## 1 Introduction

The prominence of the malar region makes the zygoma a key factor in the projection of the middle third of the face. Untreated or mistreated zygomaticomaxillary complex (ZMC) fractures may lead to functional and aesthetic defects. Delayed treatment of an old ZMC fracture usually requires releasing osteotomies (refracture) as a preceding step for the repositioning of the freed zygomatic segment. This requires the separation of the zygoma from the maxilla, temporal bone, sphenoid bone, and frontal bone. The definition of an old ZMC fracture is debatable and varies from weeks to months in the literature (1–3). The surgical approach used for refracturing old ZMC fractures usually requires coronal, lower eyelid, and intraoral incisions (4).

Before the era of virtual surgical planning, the main challenge in secondary surgical correction of old ZMC fractures was the proper repositioning of the refractured part. Lack of occlusal guidance on the one hand and the ongoing remodeling process of the fracture lines on the other hand, makes anatomical reduction difficult (5). The emergence of computer-aided design (CAD) and computer-aided manufacturing technology made it possible to virtually plan the correct anatomical position of the refractured part. Mirroring of the unaffected side onto the fractured side is a common virtual method assisting in proper symmetrical repositioning (5). The printing of the 3D planned result as a stereolithographic model can be used for the pre-surgical adjustment of fixation plates (6, 7). Polylactic acid materials can be used for printing cheap cutting guides and guiding templates to direct intraoperative osteotomies and repositioning (8, 9). Intraoperative navigation system combined with a CT can further facilitate the accuracy of the final result and is becoming a standard of care, although costs are still high (4, 6). Finally, we believe that the most advanced application of 3D technology in secondary treatment of ZMC fractures nowadays should be the designing and manufacturing of patient-specific implants (PSI) and surgical guides for achieving precise stable repositioning.

## Materials and methods

**Figure 1:**
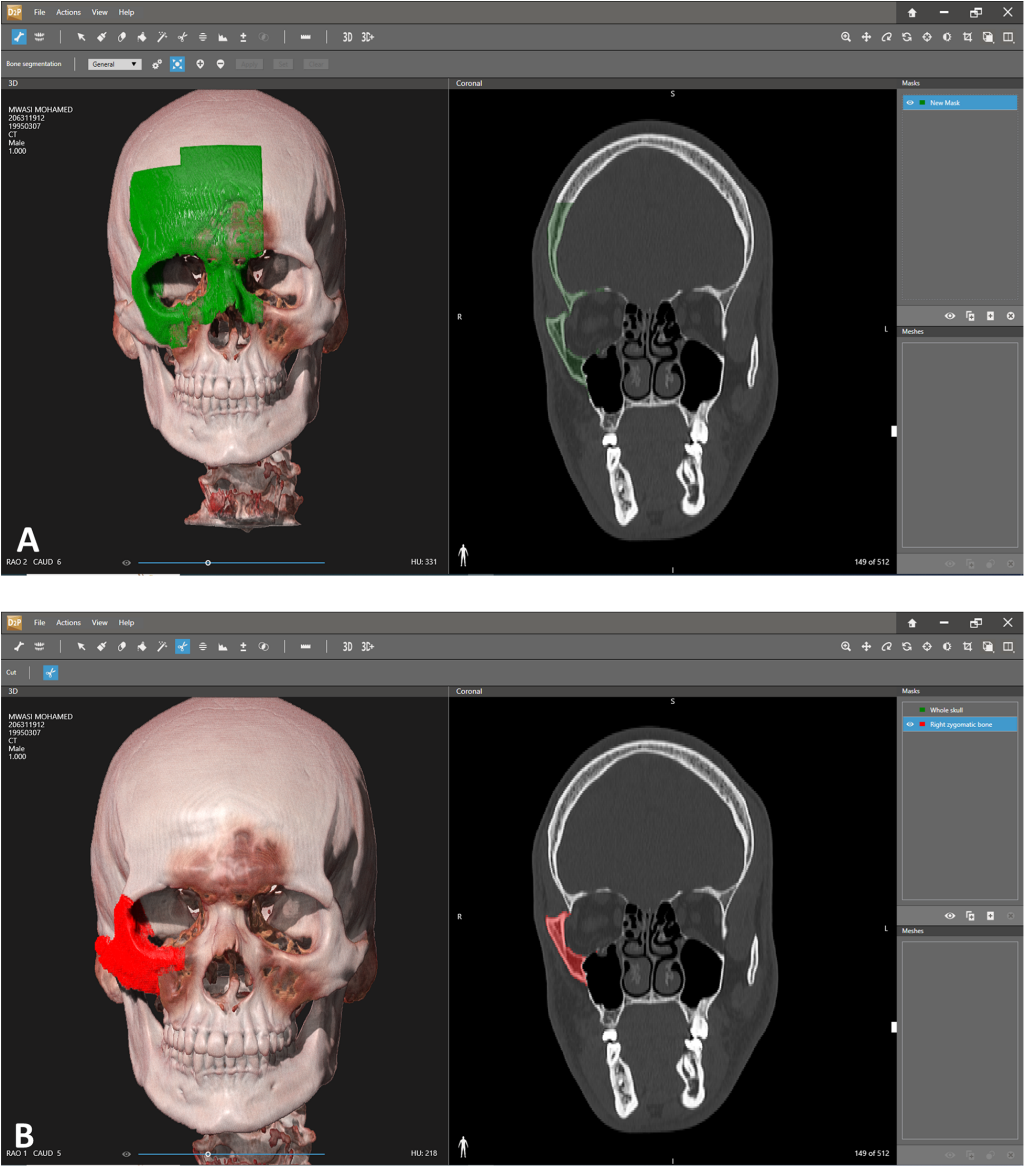
Segmentation. **(A)** Segmentation of the area of interest. Each click adds additional volume of bone to the segmented area. Notice the 3D view on the left and the coronal view on the right which allows a more precise overlook of the exact segmented area. **(B)** Segmented right zygoma turned into a mask. The next step will include turning the zygoma into a mesh and then exporting as an stl file for further manipulation in the 3D design software.

**Figure 2:**
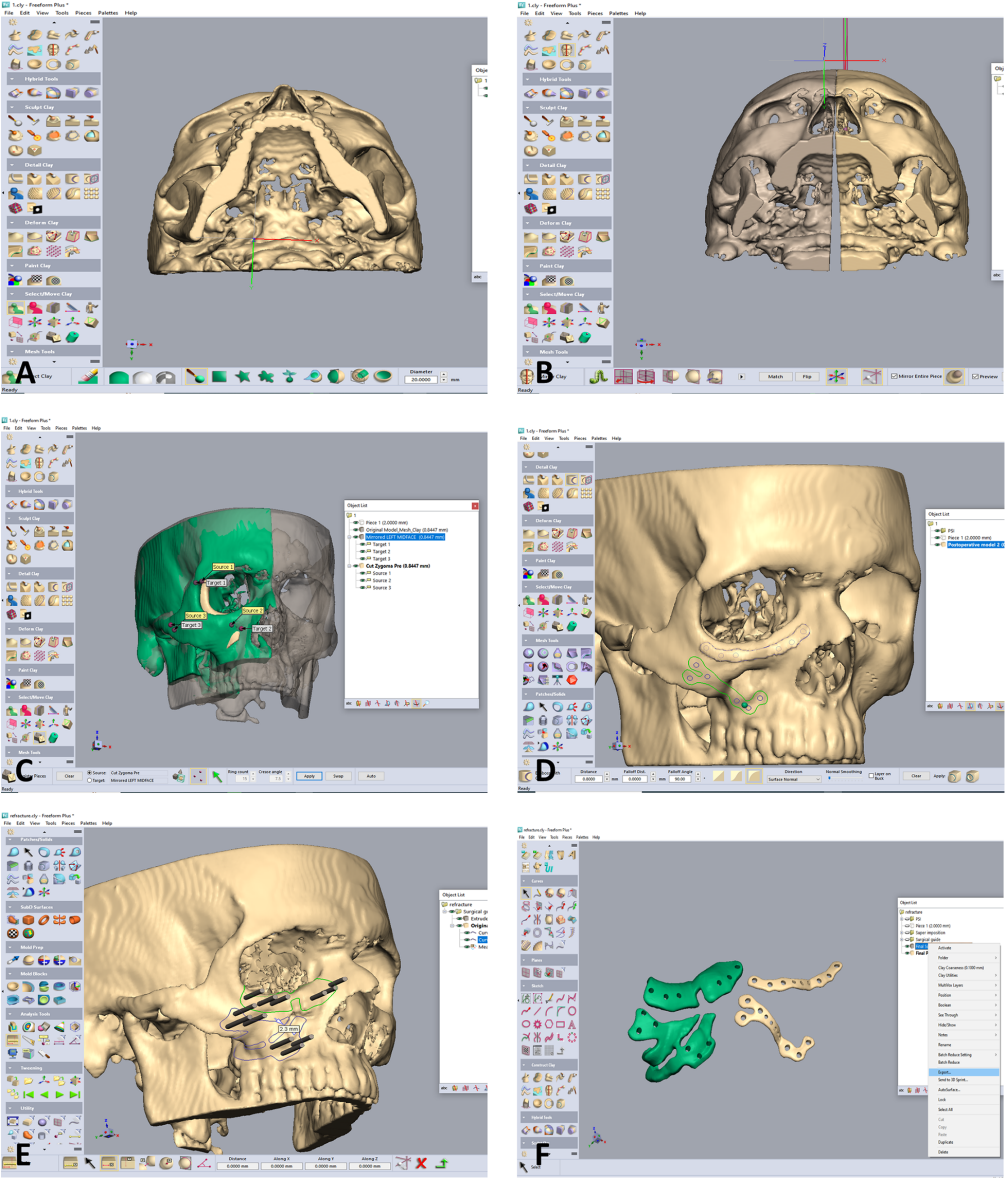
Designing of the zygoma repositioning, surgical guides and PSI. **(A)** Following segmentation into an stl file, the design is commenced using he Geomagic software. The file is imported into the software. Notice the depressed right zygoma compared to the left side. **(B)** The left side is segmented for using the mirroring technique to properly align the depressed right zygoma. **(C)** The depressed right zygoma is superimposed to the mirrored left zygoma automatically using registration of several points. **(D)** Two PSI are designed for fixation in the final aligned position. **(E)** Two surgical guides are designed, both for guiding the anterior osteotomy and for marking the position of the drill holes for the final position of the PSI. Holes drilled through the guides will match the holes of the PSI only following accurate repositioning of the zygoma according to the 3D preplanned location. **(F)** The Two surgical guides (green) and PSI's.

Retrospective study of patients suffering from a misaligned malar bone due to unsuccessful primary reduction, treated secondarily in our institute between the years 2022–2023 using 3D surgical guides and patient specific implants. The main objectives of the study were establishing a 3D based method for refracturing and repositioning the zygoma in a minimally invasive approach and assessing the accuracy of the method. Virtual planning was performed using two CAD software, the first for segmenting the area of interest turning it into a 3D STL file (Figure 1) and the second for malar bone repositioning, designing surgical guides and PSI planning. Segmentation was performed using the D2P (DICOM to Print) software (3D systems, OR, USA). 3D planning was performed using the Geomagic Freeform (3D systems). The main challenge when virtually planning these cases is the assurance of precise surface adaptation of the PSI to the refractured and repositioned malar bone in the new spatial position. To meet this essential condition, we performed a reverse sequence of planning, first designing the PSI on the repositioned refractured malar bone segment and then designing the surgical guides. Drilling holes for PSI fixation both on the non-moving maxillary bone and on the repositioned zygomatic segment are determined in the first step, based on the final planned position of the refractured malar bone. Two types of drilling holes exist—holes on the moving segment and holes on the stable part of the skeleton. Next, the repositioned zygomatic segment is superimposed back to the original pre-planned position. This backward transposition is performed along with the drilling holes of the moving segment, by keeping the same spatial relation between the drilling holes and the zygomatic segment. Next, when the malar bone with the drilling holes is properly superimposed to the pre-planned position, a drilling guide is designed to mark all hole locations both on the moving segment and on the stable skeleton (Figure 2). In Figure 2, the holes drilled through the guides will match the holes of the PSI's only following accurate repositioning of the malar bone segment, thus making the PSI a surgical guide as well as a fixation plate. The guides also serve as cutting guides for the preplanned osteotomy, separating the zygoma in an intra-oral approach, sparing the need for a coronal incision. The average time for the segmentation process, the realignment, surgical guides preparation and designing of a patient specific plate was 3 h. 3D analysis for comparing the pre-op position, planned position and post-op position of the zygoma at the bony level, was performed using CloudCompare (Open source, Freeware). This software uses the Hausdorff distance algorithm for comparing the distance between two cloud sets. This method for 3D evaluation was described in our previous article (10). For evaluating the soft tissue, a 3D PLY file was obtained using a soft-tissue scanner manufactured by Cherry Imaging Ltd (Yokneam, Israel), followed by subsequent analysis of the 3D file using the CloudCompare software.

## Results

Three patients with previous unsuccessful operations for reduction of a zygomatic fracture and who underwent a second operation utilizing 3D technology were evaluated. All patients exhibited unilateral malar depression pre-op with major improvement following treatment.

**Figure 3:**
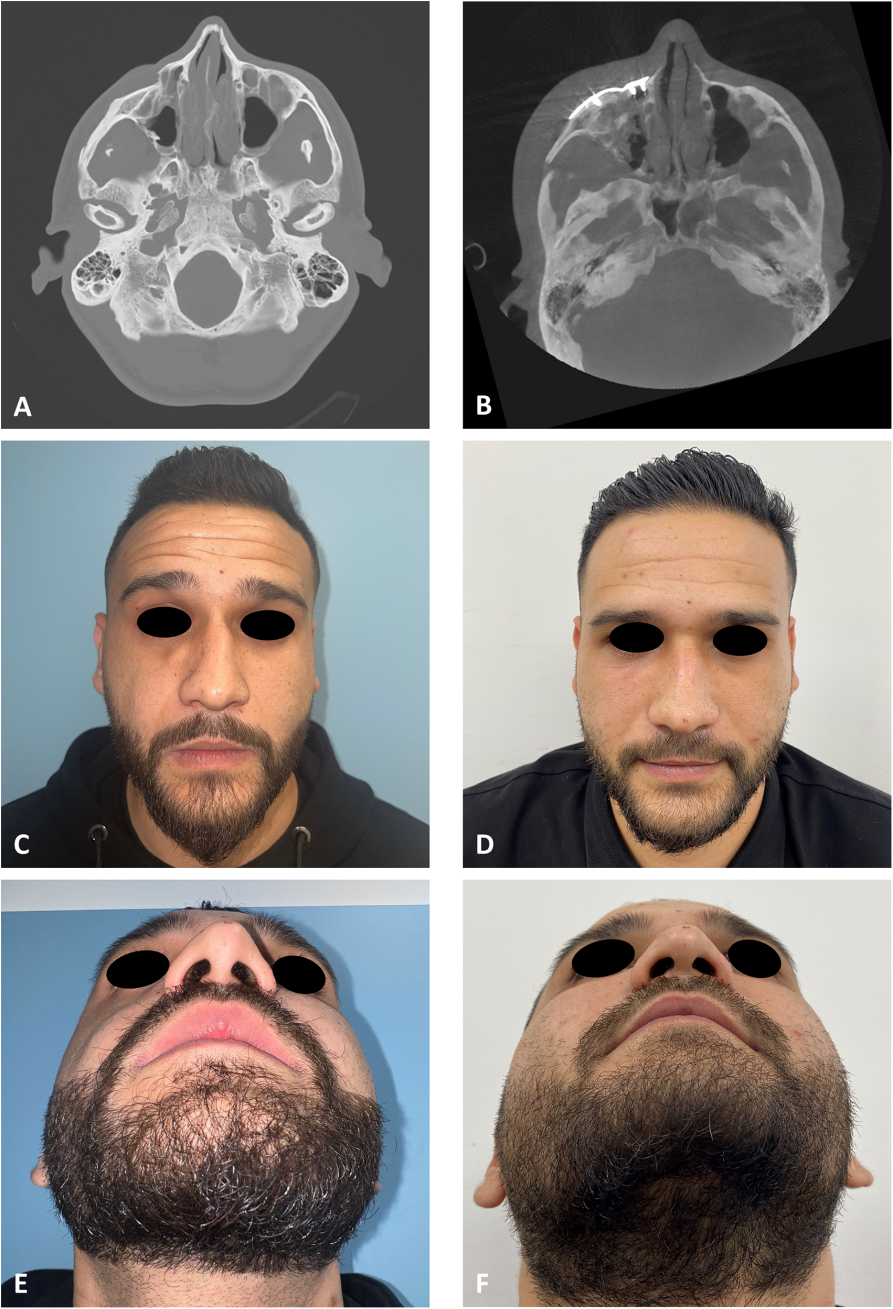
Clinical and radiological results. **(A)** CT stacking of a preoperative axial view demonstrating a depressed right zygoma. **(B)** CT stacking of a postoperative axial view demonstrating a leveled right zygoma. **(C)** Clinical preoperative en face view emphasizing the step deformity in the infraorbital rim. **(D)** Clinical postoperative en face view. **(E)** Clinical preoperative worm view emphasizing the depressed right zygoma. **(F)** Clinical postoperative worm view.

The first stage in the planning sequence was segmentation of the zygoma (Figure 1). Figure 2A demonstrates the depression of the right zygoma following the first surgery. Figure 2B shows the desired position of the right zygoma based on a mirroring technique using the left zygoma as the template. Figure 2C shows the repositioning process of the segmented depressed zygoma to the template. Figures 2D,E show the PSI's and cutting guides preparation respectively. The holes in the cutting guides fit the implants and thus guide the zygoma to the new position. The final guides and PSI's are observed in Figure 2F. Pre and post-op axial views of stacked CT slices are presented in Figures 3A,B respectively. Notice the symmetry achieved using this method. Figures 3C,D demonstrate the clinical en face change pre and post-op respectively. The major step in the inferior rim is easily identified in the pre-op photo and is diminished in the post-op photo. Pre and post-op worm views are shown in Figures 3E,F respectively emphasizing the change in malar projection.

**Figure 4:**
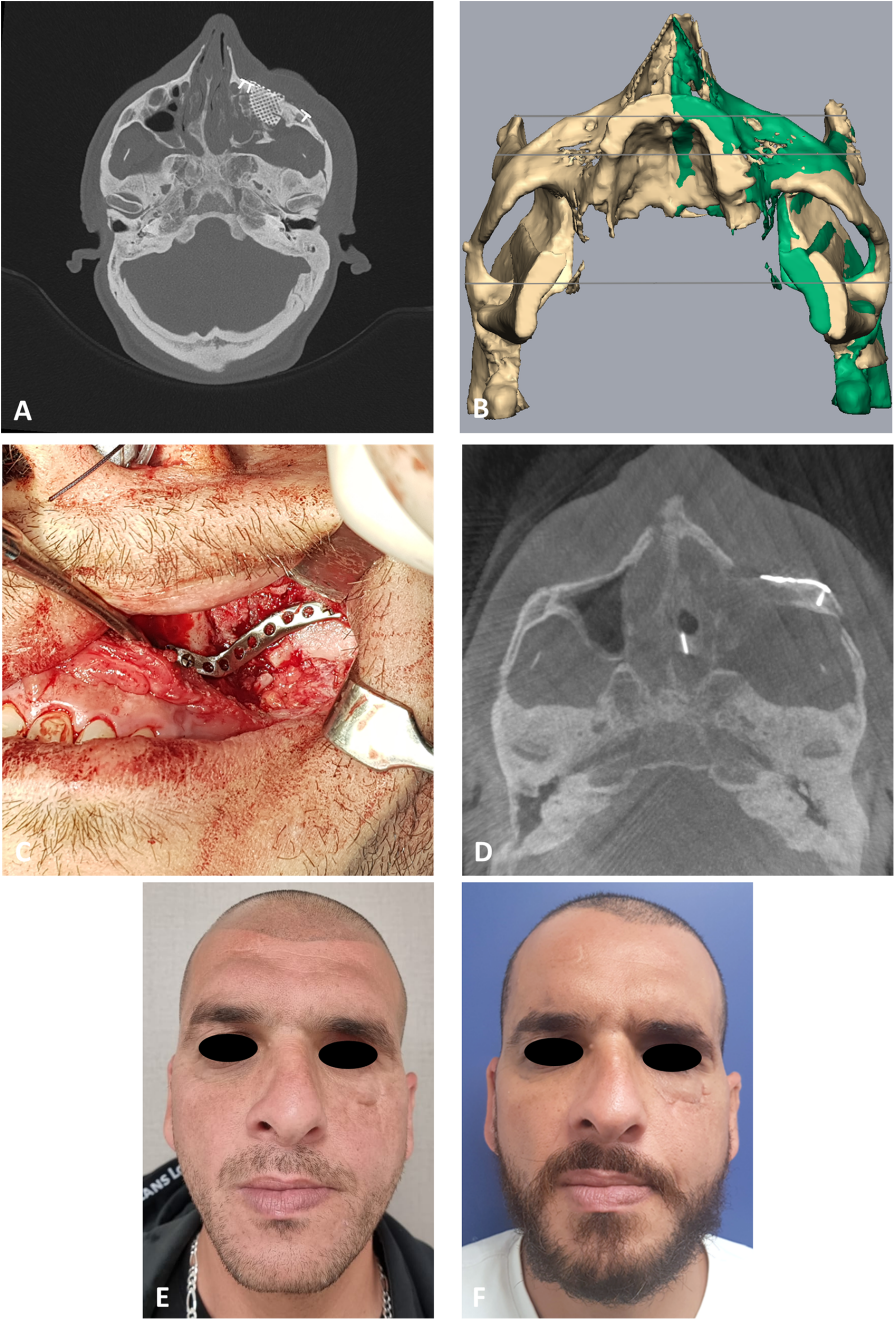
Second case presentation. **(A)** CT stacking* of a preoperative axial view demonstrating the depressed zygoma on the left following an unsuccessful surgical intervention. * Overlaying multiple CT scans into a 2D image. **(B)** Mirroring (green) of the right uninjured side to the left injured side. Mirror image is then used as a guide for the desired position of the left zygoma. **(C)** Intraoperative, intraoral view of the PSI fixating the refractured and repositioned left zygoma. **(D)** CT stacking of a intraoperative axial view demonstrating the leveled malar points following fixation with the PSI. **(E)** Clinical preoperative en face view following the first surgery demonstrating the depression of the left malar point. **(F)** Clinical postoperative en face view following the second 3D guided refracture and repositioning surgery. Notice the reconstructed malar point.

The same method is applied on the patient presented in Figure 4. Figure 4A demonstrates the depression of the left zygoma following the first surgery. Figure 4B shows the desired position of the left zygoma based on a mirroring technique using the right zygoma as the template. Clinical placement of the PSI can be observed in Figure 4C and intraoperative axial CT view shows the final position in Figure 4D. Figure 4E demonstrates the pre-op and Figure 4F the post-op clinical photos of the patient. One can appreciate the clinical volume change in the left malar and inferior orbital rim on the post-op photo.

**Figure 5:**
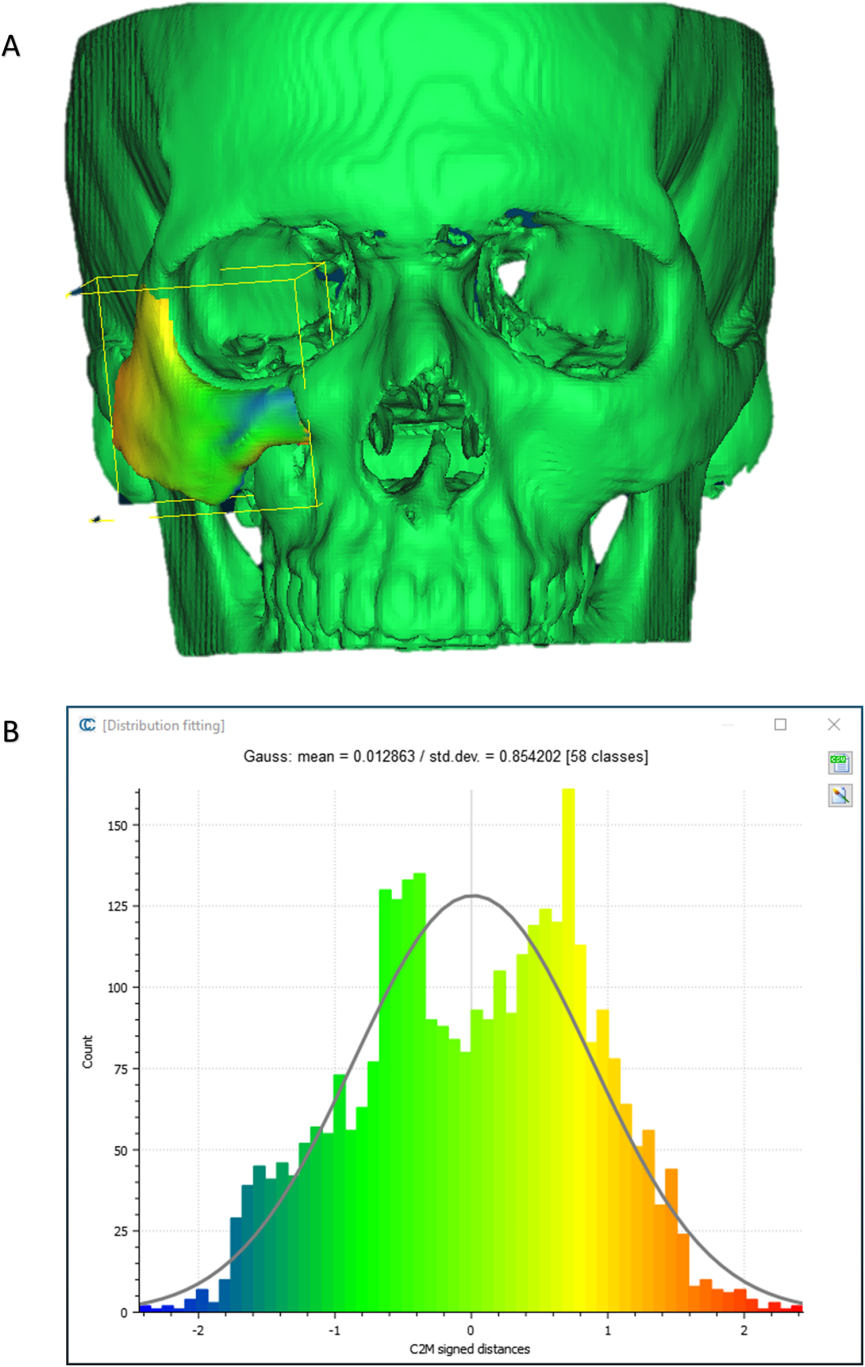
Planned vs. post-op. **(A)** Heat map demonstrating differences between a 3D reconstruction of the post-op CT compared to the Pre-op planned position of the zygoma. Blue—medial position, Orange –outward projection. **(B)** Distribution of the distances between the Post-op and Planned position of the zygoma. *Y*-axis is the number of points on the cloud-mesh software, *X*-axis is the distance of points on the post-op CT from their counter part on the planned position.

**Figure 6:**
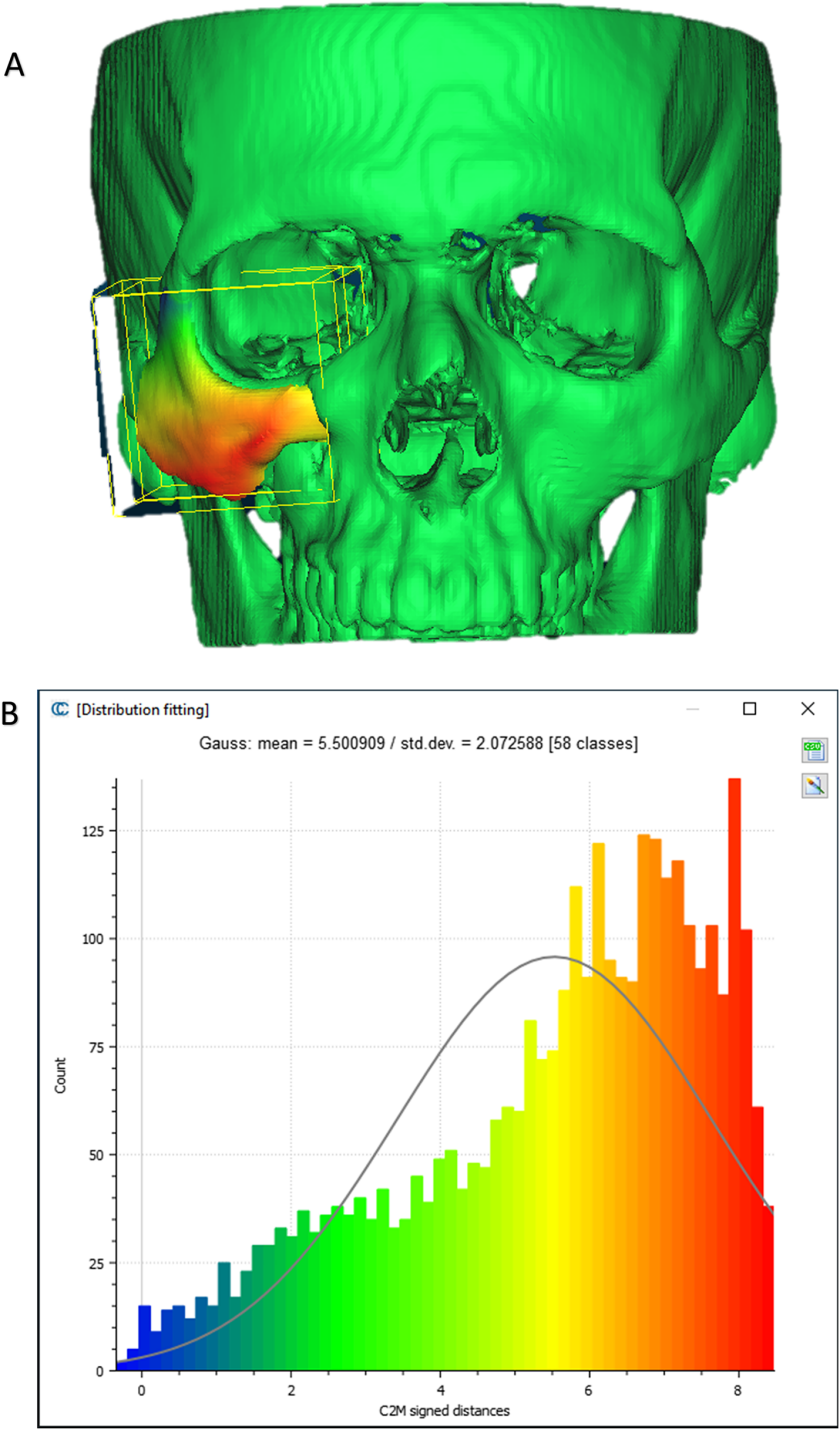
Pre vs. post-op. **(A)** Heat map demonstrating differences between a 3D reconstruction of the post-op CT compared to the Pre-op position of the zygoma. **(B)** Distribution of the distances between the Post-op and pre-op position of the zygoma. *Y*-axis is the number of points on the cloud-mesh software, *X*-axis is the distance of points on the post-op CT from their counter part on the pre-op CT.

**Figure 7:**
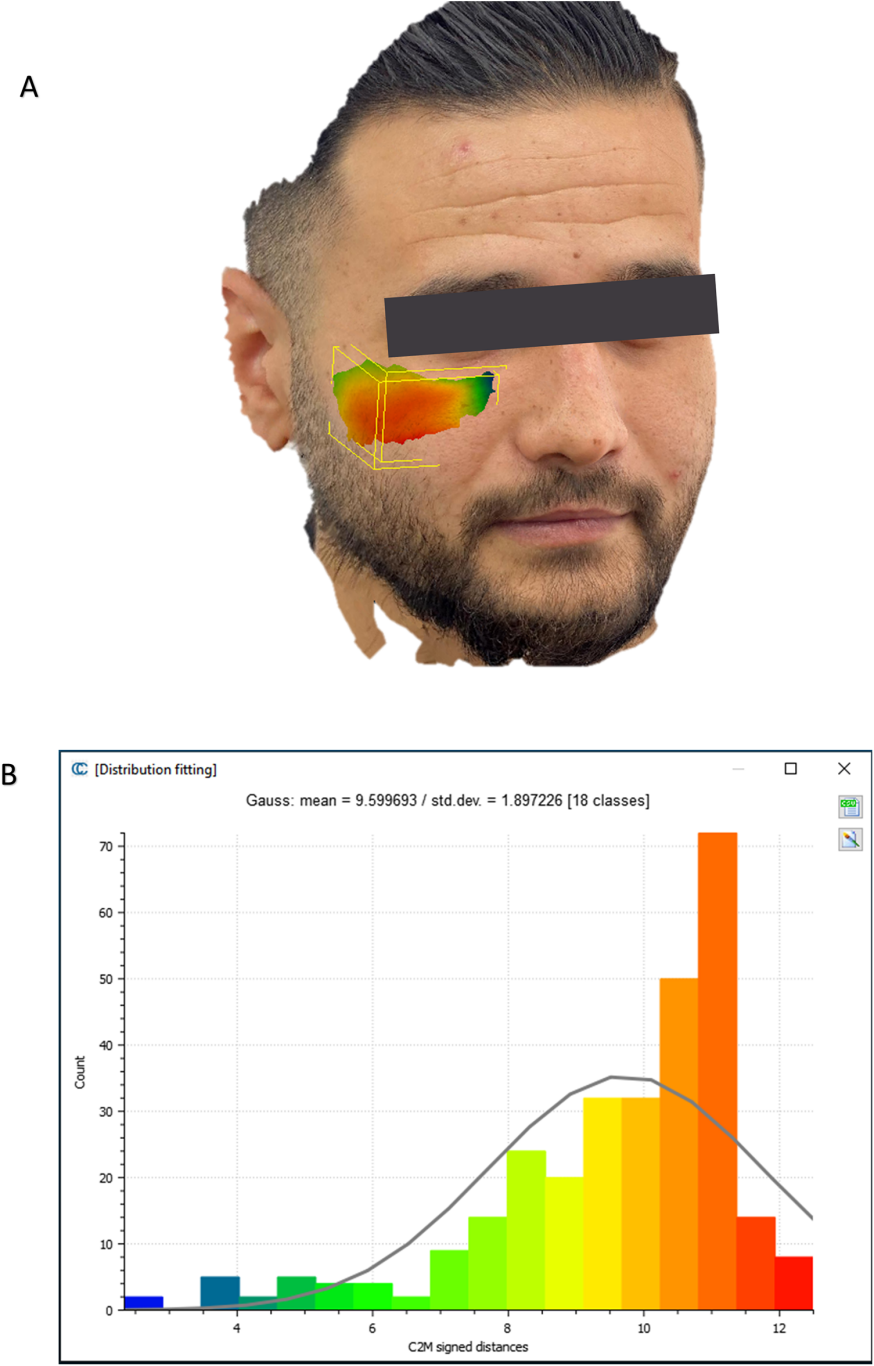
Soft tissue changes analysis. **(A)** Heat map demonstrating differences between a 3D reconstruction of the scanned post-op soft tissue and Pre-op scanned soft tissue. **(B)** Distribution of the distances between the Post-op soft tissue position and pre-op soft tissue position in the malar region. *Y*-axis is the number of points on the cloud-mesh software, *X*-axis is the distance of points on the post-op soft tissue position from their counter part on the pre-op scan.

**Figure 8:**
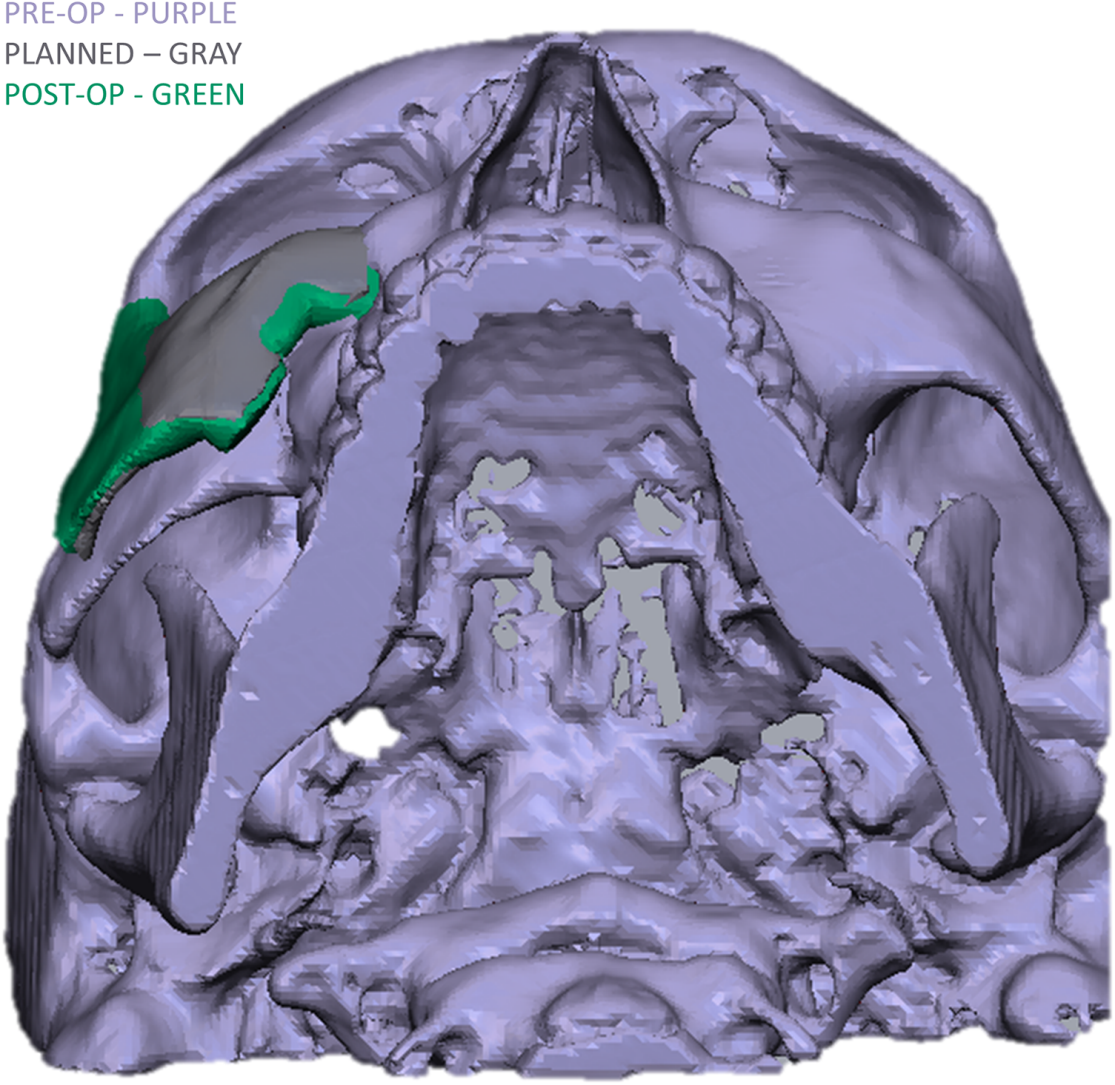
Comparison of the 3D reconstructed zygoma position in the pre, planned and post-op CT's.

**Figure 9:**
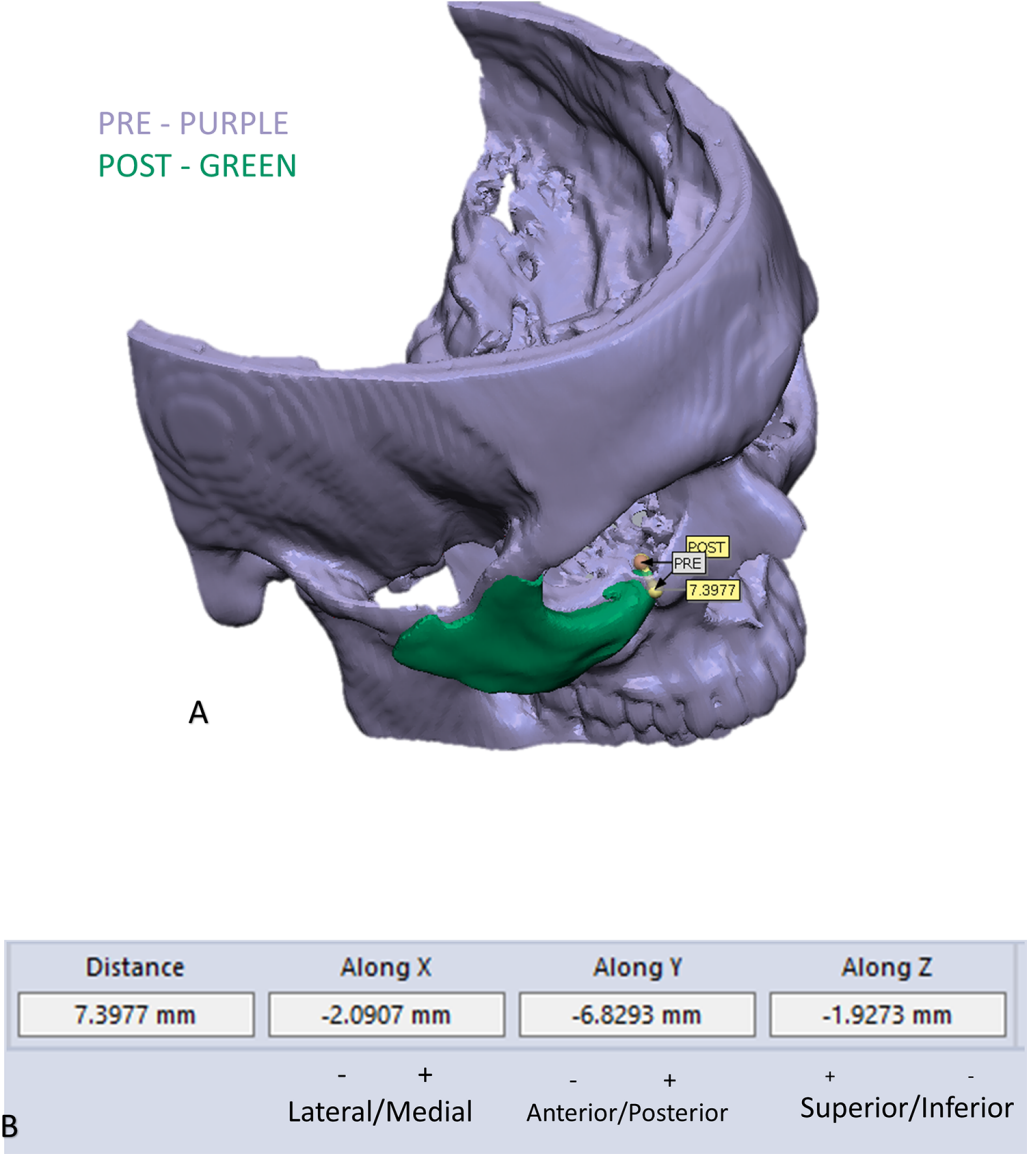
Example of measuring changes in the position of the infra orbital rim: **(A)** demonstration of the change in position of one point in the infra orbital rim, using the pre and post 3D reconstructed models. **(B)** Measurements of the movement on the three axes and the absolute distance.

To evaluate the accuracy of the method in translating the virtual plan to practice, a method for superimposing the planned and post-op location of the malar bone was applied using the CloudCompare software. A 3D heat map demonstrating the superimposition can be observed in Figure 5A. Figure 5B demonstrates the results of the superimposition using a histogram. The mean difference between the planned and post-op position was 0.72 mm, mainly in the anterior and posterior regions of the malar bone. Figure 6 presents the post-op position of the malar bone compared to the pre-op position. The 3D heatmap can be observed in Figure 6A and the histogram showing a mean difference of 5.5 mm in Figure 6B. Figure 7 shows the heatmap of the changes observed at the soft tissue level between the pre-op and post-op positions of the malar bone. Superimposition of the pre-op, planned position and post-op position is presented in Figure 8. Figure 9A demonstrates the movement of a single point in the infra-orbital rim using the pre-op and post-op 3D reconstructions. Figure 9B shows the movement of the point on the three axis as well as the absolute distance achieved.

No complications were observed in any of the patients. The mean hospital stay for the patients was three days.

## Discussion

Patient specific treatment is the future of medicine. The most famous field researching and discussing patient specific treatment is oncology. Targeted, genomic based, therapy for cancer is heavily researched (11). Patient specific treatment in surgery means planning a surgery based on imaging. There are four levels of surgical planning for a surgery. The first being simple printing of a model representing the current position and planning a surgery based on it (12). The second level includes manipulation of the current situation, such as fracture reduction and then printing the result allowing for plate bending and preparation for the surgery (13). Third level includes planning of surgical guides such as templates for proper reduction or cutting guides for resections (13). The fourth and highest level includes the planning and printing of patient specific implants. This allows for highly accurate positioning, reconstruction and reduction (10, 14–16). In this manuscript we described using the fourth level of 3D planning for the repositioning of a displaced malar bone. This is performed using surgical guides for both proper osteotomy and drill holes for accurate repositioning followed by creating patient specific implants allowing for the final fixation. The use of a surgical guide for performing the posterior osteotomy at the level of the zygomatic arch allows for an accurate intra-oral approach which spares the need for an extensive unaesthetic coronal incision. Another factor contributing to the sparing of the coronal approach is the accurate repositioning of the malar bone using the PSI. Repositioning this bone in a proper spatial position without a PSI will require extensive exposure. The same method detailed here can be applied for treating other malposition bones in the body, in the fields of orthopedics, maxillofacial surgery, ENT, plastic surgery and neurosurgery. Following the application of the method, a proper analysis method for evaluating the method had to be developed. Evaluation was performed using the CloudCompare software after manipulation of the malar bone and exporting the outer shell to avoid misinterpretation. This method was applied for evaluating superimposition of both bone and soft tissue using the Hausdorff distance algorithm. Results show high compatibility between the 3D planned position compared to the final fixated position using a 3D spatial analysis, exhibiting a 0.72 mm difference mostly at the anterior and posterior articulations of the segmented bone (Figure 5). It should be noted patient specific plates were only prepared for one articulation between the repositioned malar bone and the surrounding stable bone, while as we know four articulations exist. If another articulation would be fixated using a PSI, it is most likely accuracy would even be higher. In some of these cases several operations may have been previously performed and bone might be missing, especially at the anterior maxillary sinus wall, and thus a larger plate is designed. This may result in discomfort for the patient if the edge of the plate is located at the malar prominence. Do remember PSI pates are stronger than normal plates since they do not undergo manipulation, thus try to avoid creating thicker plates, even when bone is missing. Having said that, do not position the screw holes too posteriorly on the zygoma as the surgical access will be limited do to retraction of the soft tissues in an oral approach. One should take notice to balance between the aim of restoring the malar point while avoiding enlargement of the orbital volume. Enlargement can occur in the sphenozygomatic area due to previous comminution and can be further aggravated when rotating the segment to emphasize the malar point during the planning process. One should avoid using the anterior maxillary sinus wall as a fixation point for the PSI. Since the 3D spatial position of the zygoma is crucial, using two PSI's for fixation in the new position will result in a more accurate position compared to one PSI. Be sure to avoid the infraorbital nerve when designing the segmentation in the anterior region, this requires an accurate segmentation of the infraorbital canal.

The cases described here were planned in-house by the treating surgeons using software described in this report. It is possible to use external companies for the planning, with the associated advantages and disadvantages. Using external companies increases the price significantly, delays treatment and the surgeon is less prepared as he is not closely acquainted with any difficulties encountered by the specific anatomy. On the other hand, using external companies reduces the time spent by the surgeon for designing the case, some of the companies have extensive experience, mostly in classic surgeries such as in orthognathic cases and it spares the need for acquiring 3D designing software.

We conclude using 3D planning and PSI for secondary refracture and reconstruction of a misaligned malar bone is a highly accurate and predictable method which allows for reducing human error dramatically in these challenging cases. We think every craniomaxillofacial surgeon should be familiarized with this option, whether it is performed in-house, such as in our cases, or by outsourcing the planning.

## Data Availability

The raw data supporting the conclusions of this article will be made available by the authors, without undue reservation.
